# Interorganizational Knowledge Sharing to Establish Digital Health Learning Ecosystems: Qualitative Evaluation of a National Digital Health Transformation Program in England

**DOI:** 10.2196/23372

**Published:** 2021-08-19

**Authors:** Kathrin Cresswell, Aziz Sheikh, Bryony Dean Franklin, Marta Krasuska, Hung The Nguyen, Susan Hinder, Wendy Lane, Hajar Mozaffar, Kathy Mason, Sally Eason, Henry Potts, Robin Williams

**Affiliations:** 1 Usher Institute The University of Edinburgh Edinburgh United Kingdom; 2 School of Pharmacy University College London London United Kingdom; 3 Institute for the Study of Science, Technology and Innovation The University of Edinburgh Edinburgh United Kingdom; 4 National Health Services Arden and Greater East Midlands Commissioning Support Unit Warwick United Kingdom; 5 Business School The University of Edinburgh Edinburgh United Kingdom; 6 Institute of Health Informatics University College London London United Kingdom

**Keywords:** digital transformation, health system, learning ecosystem

## Abstract

**Background:**

The English Global Digital Exemplar (GDE) program is one of the first concerted efforts to create a digital health learning ecosystem across a national health service.

**Objective:**

This study aims to explore mechanisms that support or inhibit the exchange of interorganizational digital transformation knowledge.

**Methods:**

We conducted a formative qualitative evaluation of the GDE program. We used semistructured interviews with clinical, technical, and managerial staff; national program managers and network leaders; nonparticipant observations of knowledge transfer activities through attending meetings, workshops, and conferences; and documentary analysis of policy documents. The data were thematically analyzed by drawing on a theory-informed sociotechnical coding framework. We used a mixture of deductive and inductive methods, supported by NVivo software, to facilitate coding.

**Results:**

We conducted 341 one-on-one and 116 group interviews, observed 86 meetings, and analyzed 245 documents from 36 participating provider organizations. We also conducted 51 high-level interviews with policy makers and vendors; performed 77 observations of national meetings, workshops, and conferences; and analyzed 80 national documents. Formal processes put in place by the GDE program to initiate and reinforce knowledge transfer and learning have accelerated the growth of informal knowledge networking and helped establish the foundations of a learning ecosystem. However, formal networks were most effective when supported by informal networking. The benefits of networking were enhanced (and costs reduced) by geographical proximity, shared culture and context, common technological functionality, regional and strategic alignments, and professional agendas.

**Conclusions:**

Knowledge exchange is most effective when sustained through informal networking driven by the mutual benefits of sharing knowledge and convergence between group members in their organizational and technological setting and goals. Policy interventions need to enhance incentives and reduce barriers to sharing across the ecosystem, be flexible in tailoring formal interventions to emerging needs, and promote informal knowledge sharing.

## Introduction

### Background

Digital transformation is now central to most health system strategies, as governments around the world seek to address the challenges associated with demographic shifts and the need for sustainable care provision to aging populations with complex long-term conditions [1,2]. Although policy makers and implementers generally agree on the potential of health information technology (HIT) to improve safety, quality, and efficiency of care, strategies for procurement, implementation, and optimization vary significantly across settings [3-5]. Large-scale HIT-enabled transformation programs have met with varying success, and there is no agreed strategy on how best to achieve digital transformation at scale [6-9].

Many aspects of digital transformation have been studied [10-12]. However, interorganizational knowledge sharing is a key feature of recent initiatives to promote concerted change across multiple organizations by establishing a learning ecosystem [13,14]. Understanding interorganizational knowledge transfer may help to mitigate risks by avoiding repetition of mistakes, thereby saving money and minimizing potential threats to patient safety and quality of care. Concerted adoption might also reduce inefficiencies of fragmented one-off implementations by encouraging learning across communities of adopters and increasing their influence on system development.

We use the term *learning ecosystem* to refer to interorganizational sharing of technology, knowledge, and know-how to achieve digital transformation (ie, to change technologies and organizations). We differentiate this from the notion of *learning health systems*, which focuses on optimizing the use of clinical and operational data to advance and apply medical research (ie, to advance the clinical cycle or improve care processes) [15]. The learning ecosystem highlights that knowledge and experience of technology adoption and implementation is particularly valuable for members of other organizations contemplating similar digitally enabled transformation (as well as for vendors and policy makers) [16].

Although there have been local examples of attempts to promote digital health–related knowledge exchange, these are often not systematically evaluated and are poorly theorized [17,18]. In contrast, in the commercial sector, a large body of literature explores knowledge transfer between technology vendors and users [19-22]. This work highlights the key role of various intermediaries in bridging gaps, translating, and facilitating information flows between different stakeholder groups [23,24]. In addition to formal organizational links (eg, vendor-hosted user groups), informal networking, driven by the benefits of knowledge transfer, can be particularly important in communicating *sticky* information (information that is hard to acquire and intimately linked to its context of use) [25]. Some papers discuss user-to-user sharing of knowledge, but this focuses mainly on consumer products or open-source applications [26,27].

### Objectives

We were commissioned to conduct an independent evaluation of the Global Digital Exemplar (GDE) program. This was the first national digital transformation program designed to facilitate concerted interorganizational knowledge exchange and create a digital health learning ecosystem [28,29]. This program was set up following a national review of national HIT strategy led by Robert Wachter [30], with the National Health Service (NHS) England committing £395 million (US $544 million) to support the development and interorganizational knowledge sharing between a selected group of digitally mature provider organizations. A total of 51 organizations participated in the program, including 33 acute care, 15 mental health, and 3 ambulance provider organizations. The latter 3 were not included in the evaluation along with 9 sites whose launch was delayed, and 3 where organizations merged. As a result, the evaluation covered 36 provider organizations. Some of these were designated leaders who were to implement first (GDEs) and others partnered with GDEs to learn from and follow them (fast followers).

We sought to answer the following research question: How is interorganizational knowledge sharing taking place within the GDE program?

## Methods

### Setting

The GDE program’s attempt to establish a digital health learning ecosystem was accompanied by related national initiatives, including professional training and education [31]. Specific mechanisms to promote interorganizational knowledge transfer included the following:

1. GDE and fast follower pairings: This involved pairing digitally advanced exemplar provider organizations (GDEs) with partner organizations (fast followers) who would follow and learn from GDEs throughout the duration of the program. The rationale for the pairings varied among stakeholders, and no official documentation was available on the issue. Most organizations appeared to choose their own partners. Other pairings were established by external stakeholders. Care settings were paired with each other so that mental health organizations were matched with other mental health organizations, and acute organizations were paired with other acute organizations.
2. Establishing a series of national learning networks to promote knowledge transfer among participating provider organizations and across the wider NHS.
3. Blueprinting: This involved asking all participating provider organizations to produce documents (blueprints) to capture implementation, adoption and optimization experiences.

### Design

We conducted an independent, longitudinal, qualitative, formative evaluation of the GDE program, exploring digital transformation and knowledge transfer in participating acute and mental health provider organizations. The study period was from January 2018 to March 2020. The evaluation revolved around two core themes: first digital transformation of sites, and second, the processes of interorganizational knowledge transfer explored in this paper. Some findings have been reported in previous studies [32,33]. Our evaluation reports are available on our website [29].

Methods included a combination of in-depth semistructured one-on-one and group interviews with relevant organizational stakeholders (managers and clinicians), documentary analyses of organizational strategic plans, and ethnographic fieldwork (nonparticipant observations of strategic meetings and site visits) to explore national knowledge networks and linkages between organizations. This allowed insights into local knowledge, organizational context and progress, and formal and informal knowledge transfer mechanisms as experienced by those participating in knowledge transfer activities. We also collected a range of national documents addressing planned knowledge transfer mechanisms, observed national workshops and conferences where knowledge was shared formally and informally, and conducted in-depth interviews with national program managers and system vendors to gain insights into how they planned and participated in knowledge sharing.

Provider organizations were conceptualized as case studies [34]. We conducted in-depth studies of 12 organizations (A-M) and broader case studies of the remaining 24 organizations. In-depth case studies were selected for maximum variation, including different core technological systems, geographical locations, organizational types (acute and mental health), and baseline levels of digital maturity. Detailed methods of our full evaluation, of which the creation of a learning ecosystem was a central theme, are described in our study protocol [35].

### Analysis

We combined inductive and deductive methods, drawing on a theory-informed, sociotechnical coding framework [36]. Lead researchers (SH, MK, and HTN) initially analyzed knowledge flows in in-depth case studies and then tested and validated these emerging findings against broader case studies. We then integrated developing narratives with accounts of the wider macroenvironmental landscape from policy, commercial, and independent stakeholders and tested these against case study data [37]. Narrative accounts were produced collectively and through discussions in team meetings, where we paid most attention to emerging tensions and conflicting findings. We have published detailed empirical accounts of the operation of individual learning components initiated through the program elsewhere [32,33]. The current analysis focused on integrating different strands of inquiry relating to knowledge networks across the GDE program and within the wider macroenvironmental context. In doing so, we also identified the roles and effectiveness of various intermediaries. We used the COREQ (Consolidated Criteria for Reporting Qualitative Studies) guidelines [38].

### Ethical Approval

This work received institutional ethical approval from the School of Social and Political Science at the University of Edinburgh, UK.

## Results

### Overview

We conducted 341 one-on-one and 116 group interviews, observed 86 meetings, and analyzed 245 documents from 36 participating provider organizations (Textbox 1; Table 1; Table S1 of Multimedia Appendix 1). We also conducted 51 high-level interviews with policy makers and vendors; 77 observations of national meetings, workshops, and conferences; and analyzed 80 national documents.

**Study data set.**

**Data Collected in the In-depth Case Study Sites (Global Digital Exemplar [GDE])**

- 12 provider organizations
- 8 GDEs: 6 acute and 2 mental health
- 4 fast followers: 3 acute and 1 specialist fast follower
- 224 one-on-one interviews
- 67 group interviews
- 104 documents
- 67 meetings observed

**Data Collected in the Broad Case Study Sites**

- 24 provider organizations
- 15 GDEs: 10 acute and 5 mental health
- 9 acute fast followers
- 117 one-on-one interviews
- 49 group interviews
- 141 documents
- 19 meetings observed

**Data Collected Elsewhere**

- 51 high-level interviews with policy makers and vendors
- Nonparticipant observations of 77 national meetings, workshops, and conferences
- 80 documents

**Table 1:** Description of the sample in the wider Global Digital Exemplar Program landscape (n=48)^a^.

Overall	Included in in-depth studies (n=12), n (%)	Included in broader studies (n=24), n (%)	Omitted because of late admission to program (n=9), n (%)	Omitted because fast follower merged with GDE^b^ (n=3), n (%)	Total (n=48), n (%)
Overall number of GDEs (excluding ambulance GDEs): 16 acute and 7 mental health	8 (66)	15 (63)	0 (0)	0 (0)	23
Overall number of fast followers: 17 acute and 8 mental health	4 (33)	9 (38)	9 (100)	3 (100)	25

^a^The number of overall Global Digital Exemplars and fast followers differ from those included in our study, as there were some mergers and delays in start dates, which meant that we did not include some provider organizations.

^b^GDE: Global Digital Exemplar.

In our broader sample, 19 pairings of GDEs and fast followers had a common core system, and 15 organizations were in the same local strategic groupings coordinating collaborations of health care organizations and local authorities (including so-called sustainability and transformation partnerships and integrated care systems). These local strategic groupings developed in parallel with the program. In our 12 in-depth case studies, 6 pairings were located in the same local strategic grouping, and 10 had the same core system as their fast follower.

**Figure 1:**
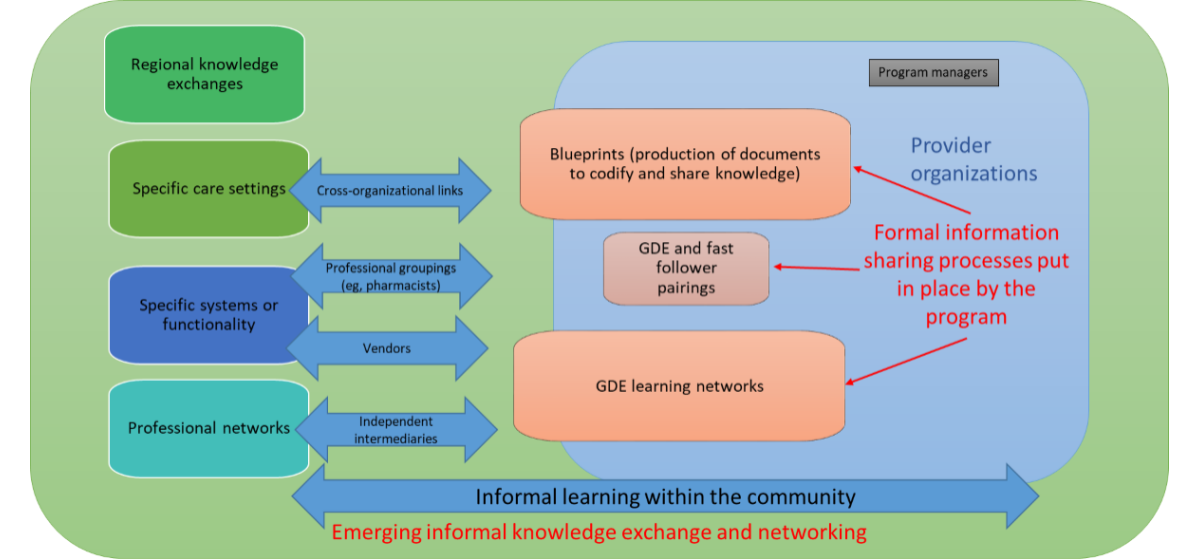
Formal and informal learning and knowledge exchange processes in the Global Digital Exemplar program. GDE: Global Digital Exemplar.

Figure 1 illustrates the emerging formal and informal learning and knowledge exchange processes, knowledge exchange forms, and key intermediaries in the program. We use the term *formal* to describe knowledge exchange processes resulting directly from planned program activities, including those emerging from GDE and fast follower relationships, blueprinting of documents, and program learning networks. We use the term *informal* to describe emerging knowledge exchange processes either as an unanticipated, indirect consequence of these activities or as unrelated activities.

Overall, our work suggests that GDE initiatives, coupled with the broader impetus generated by the program, have promoted a burgeoning learning culture across digitally engaged provider organizations and GDE and fast follower pairs, with increased sharing of knowledge and experience. All but 5 provider organizations in our sample described involvement in networking activities, sharing knowledge and experience, and learning from others. We also observed some evidence of the emergence of a learning ethos in the NHS reinforced by these processes:

...[W]e’re starting to share what we’re doing, in a demonstrable way, and start to see it, and it was quite powerful.

Site 14, nonclinical digital leader, broader case study

[Provider organization] had spent about a year building pediatric medicines. And they said here, you can have it. So that’s a year’s work, that’s non-trivial. They just simply gave it to us. Now would that have happened two years ago? Three years ago?...So there are people sharing things of real value, real cost, real-time...which is excellent. So are we creating new knowledge by that, I’m not sure. Are we sharing and optimizing that knowledge? Very definitely.

Site L, chief information officer, in-depth case study

### Evolving Formal Processes to Promote a National Digital Health Learning Ecosystem

#### Evolving Formal Processes

Program managers implemented several linked formal initiatives to facilitate knowledge transfer within tight time frames. Formal mechanisms were encouraged and strongly supported by the burgeoning of informal networking and sharing of knowledge and experience. These developments led to changes in the strategic focus of the program. In particular, the strategy associated with the production and distribution of blueprints evolved to become a key component of the learning ecosystem. The blueprinting process changed as the user community (provider organizations) became actively engaged in developing the mechanisms for their production, distribution, and use. Blueprints were initially conceived as repositories of the extensive information needed for the rapid procurement and implementation of validated technologies that could then be widely disseminated. However, provider organizations found them useful in unanticipated ways—as an initial introduction to a topic and as a way to identify and make contact with people involved in implementations—leading to email exchanges, phone calls, and site visits. Thus, blueprinting changed from an activity of capturing digital transformation knowledge in artifacts to a means of facilitating informal networking:

[Blueprinting]’s supposed to be not just about taking and adopting, it’s to open up conversations.

Site 8, nonclinical digital leader, broader case study

The evolving blueprinting concept also saw a relaunched web-based platform, radically reconceptualizing blueprints as *a structured collection of knowledge assets and associated methodology for using them* [39]. It largely overtook centrally driven GDE learning networks; however, where professional groups drove learning networks and where networks tackled specific functionality, these were very successful in attracting and sustaining participation and became national communities of practice. Occupational groupings that aligned their professional interests with enhancing practice through digital transformation were particularly successful. For example, pharmacists were actively involved in knowledge networks around hospital electronic prescribing and medicines administration systems:

...[A]ll the GDE groups that work on prescribing, we’re having monthly phone calls and meetings

Site H, senior manager, in-depth case study

We observed that national activities in many instances helped to initiate and sustain informal networking. Where informal networking emerged, it maximized the effectiveness of formal interorganizational knowledge transfer processes and ensured their sustainability:

...[N]othing is really very formal any more, they will pick up the phone and phone [other GDE] and ask how they are doing it. So, it’s those informal relationships that I think are really beneficial.

Site B, GDE program staff, in-depth case study

#### Contextual Factors Influencing Informal Knowledge Sharing

##### Contextual Factors in Provider Organizations

Although informal processes constituted a large and effective part of knowledge transfer and networking, these varied significantly among participating provider organizations. Analyzing these differences provides insights into facilitators and barriers to knowledge transfer.

We found that the adoption of a common core system (such as for electronic health records and hospital electronic prescribing and medicines administration systems), prior relationships, geographical proximity, and regional alignment were, in most instances, beneficial for knowledge sharing and networking by reducing the costs of establishing and maintaining interactions.

Participants also frequently mentioned having similar organizational ethos and culture and similar (or the same) patient populations as facilitators:

...[W]e’re a similar size as [organization] with a similar footprint of patients with similar economic and geographical pressures, so that’s really helpful.

Site C, chief nursing informatics officer, in-depth case study

Conversely, differences between organizations in culture, patient populations, and needs were seen as barriers to knowledge sharing.

##### Common Challenges and Technological Functionality

Similarities between organizational settings reduced learning costs and increased the relevance and benefits of knowledge exchange. The common challenges faced by specific care settings were also facilitators of informal interorganizational networking and knowledge transfer. For instance, we observed productive knowledge exchanges among mental health providers. These shared specific needs and purposes (that might be overlooked by larger acute hospitals) and began to organize informal collaboration.

Common technological functionality was a key facilitator, as organizations with the same vendor often faced similar challenges and sharing of lessons could contribute to avoiding repeating mistakes. There was also scope to transfer detailed elements of system configuration, removing the need to replicate onerous coding work and speeding up implementation:

...[T]hat has been happening...the knowledge sharing, especially in those organizations with similar systems, oh, you’ve just done that, so we’ll go and look at it, you’ve done that, we’ll take this.

Site G, senior manager, in-depth case study

Clinically, I think it’s fantastic, and organizationally and operationally with [GDE], because you’ve got the same system and we’re taking a lot of their content that they’re developing and then we copy it.

Site B, chief medicines information officer, in-depth case study

The GDE program further encouraged links between users and vendors, including the development of user groups around major system suppliers. In some instances, organizations reported increased leverage over system vendors and joint procurement:

...[W]orking with other GDEs has...given us a bigger voice to talk to suppliers, it’s given us an opportunity to introduce new people into the market, and then share that experience with others.

Site F, chief information officer, in-depth case study

##### Reputational Benefits and Competition

The reputational benefits of GDE membership were an important motivator for knowledge sharing, but provider organizations were in some respects competing for status and resources, which inhibited knowledge sharing. In particular, some fast follower organizations were unhappy to be designated as *followers*, especially where they felt they possessed, or would soon attain, greater capability than their GDE:

I don’t call this fast follower I like the word partner...I think that some of the work that we’re doing we’re leading rather than following our GDE.

Site B, information management and technology manager, in-depth case study

One organization was concerned about reputational risk if their partner performed poorly:

I think people are worried about reputational damage. So, if the [provider organization] that you were partnered with would never ever get to a position where you were, is that a failure on the mentoring a [provider organization], or is it a failure with the [provider organization] trying to catch up?

Site A, information technology manager, in-depth case study

Some GDEs were seeking recognition as the most digitally mature provider organization in the country. Although under some circumstances, organizational status conflicts had inhibited knowledge sharing (eg, where there was a history of local competition between neighboring organizations), these were exceptions to a broader pattern whereby a culture of sharing prevailed.

### Mediators Facilitating Knowledge Transfer Across the Wider Health System

Some stakeholders acted as knowledge exchange mediators, extracting and collating lessons from particular implementations for wider applications. Here, a range of interorganizational networks facilitated knowledge exchanges between provider organizations. These included system vendors who coordinated networking among national organizations with the same system (eg, through user groups and pilot site visits, connecting key individuals to work together across organizations) and promoted connections with international organizations with the same system:

[Place in the United States] was one we met through [vendor], because they’re a [vendor] client, and [name], who’s their Chief Clinical Information Officer, came here, and again we’ve kept in touch with them.

Site 19, chief information officer, broader case study

Professional networks also played an important role. These allowed members with a common interest to get together, and to exchange ideas, challenges, and lessons learned in a neutral space.

Moreover, we observed the development of specialist digital transformation managerial communities that facilitated informal networking. An example here included the formation of an informal national network of chief clinical information officers and a range of web-based and face-to-face networking activities organized by an independent community of digital health professionals [40]:

There’s an outfit called Digital Health Networks...and they run a series of forums...it’s an online community that’s growing all the time, and is exchanging ideas very productively.

Site C, clinical digital lead, in-depth case study

Another example was the NHS Digital Academy, a national program to develop digital health leadership capabilities in the NHS [31]. During our data collection period, 50 participants from 29 different GDE provider organizations studied at the NHS Digital Academy:

...[T]he Digital Academy has really shown that it’s phenomenally important...we’ve had loads of conversations, over dinner and things...about what they’re doing, what we’re doing...and, actually, that’s been really beneficial because otherwise we probably wouldn’t have found time to have those conversations.

Site C, clinical digital lead, in-depth case study

### Relative Costs and Efforts Associated With Knowledge Transfer

The mutual benefits of shared learning and an ethos of public health benefit facilitated emerging small-scale exchanges. The biggest barrier to knowledge transfer cited in our sample was competing demands on participants’ time, particularly given the priorities for health professionals to provide day-to-day care:

...[Knowledge transfer is] one of those things that you need to make time for and we’re all really busy in our day-to-day roles...

Site D, chief nursing informatics officer, in-depth case study

Knowledge sharing activities were particularly burdensome for organizations (mainly GDEs) that were perceived as national leaders and, therefore, had many requests from a range of other organizations to share knowledge. Those seeking to establish themselves as national leaders expressed concern that moving forward as a group of organizations could slow down processes such as procurement and thereby hold back their development:

...[T]hat’s just the difficulty of moving together as a group of organizations, even though we do work very well as a unit. It’s those sorts of things where there are more complications in terms of procurement and contracting and so on and so forth.

Site F, chief information officer, in-depth case study

Knowledge sharing through informal networking demands people’s time and offers fewer obvious opportunities for economies of scale than, for example, circulating documents. There were some concerns that the cost of networking would threaten the sustainability of sharing activities.

Individuals and organizations benefited from learning by receiving information. They could also experience reputational benefits that could improve their status and strengthen individual expert careers. Networking and knowledge transfer were enhanced when the learning costs were minimized and the benefits maximized. However, issues emerged where there was asymmetry between knowledge provision and knowledge receipt for organizations making this informal mutuality difficult to sustain. For example, this was an issue where provider organizations engaged with large numbers of adopters and where knowledge transfer took a lot of resources.

Nationally organized activities mitigated barriers to an extent by reducing the cost of knowledge transfer to provider organizations. Different types of national interventions played a catalytic role. Critical factors included stimulating discussion topics and shaping agendas, setting up webinars and knowledge transfer work, and curating artifacts for sharing:

...[W]e’ve had the capacity to go out and talk to other organizations across the UK which we’ve done...and the project team have the capacity and the ability to do that. We would never have been able to do that pre-GDE.

Site E, GDE program staff, in-depth case study

## Discussion

### Principal Findings

Our exploration of interorganizational knowledge transfer in the GDE program shows that the program has made a major contribution to the current upsurge in knowledge transfer across the NHS. The combination of formal learning mechanisms and processes to initiate a national digital health learning ecosystem promoted systemic learning; however, it was most successful when supported by informal networks. Formal knowledge transfer mechanisms did not necessarily work in a planned manner. They evolved over time and prompted a dramatic growth in informal learning among organizations and specialist communities.

### Strengths and Limitations

We conducted a national formative evaluation of a first-of-type digitally enabled national transformation program and collected a large qualitative data set from a range of settings and data sources. Our research design, combining in-depth with broader data collection, allowed us to balance the depth and breadth of insights. We achieved this by analyzing change processes and mechanisms of knowledge transfer in detailed studies of selected provider organizations, while testing these emerging findings in a wider range of settings and placing them within the national context of the program. In doing so, we gained rich insights into how knowledge transfer took place in an evolving interorganizational learning ecosystem.

However, as this work is based on a national qualitative case study, the findings need to be interpreted with caution. Our work occurred in a public managed health system, and associated values and motivations may affect generalizability to private providers. Our sample was purposive and focused on clinical leaders and managers and did not capture the perspectives of a broader range of frontline staff. This may be particularly important, considering that our findings highlight the central role of informal networking. We captured perceptions of the importance of informal channels but had limited opportunity to examine the spread and operation of networking among those at the coalface of providing care. In addition, our fieldwork examined knowledge transfer from organizations participating in the GDE program but not organizations outside the program. There is also an overall difficulty in capturing informal knowledge exchanges and a risk that attempts to monitor these will overlook important knowledge transfer processes.

### Integration of Findings With the Current Literature

Our findings add to the sparse existing empirical literature exploring learning ecosystems and interorganizational knowledge transfer in digital transformation in health care [24,41,42]. This work highlights the complexity of the health care landscape, involving multiple users and producers of knowledge, driven by various, and at times, conflicting motivations [24,41,42]. Thus, there is no recipe for successful knowledge transfer in innovation ecosystems, and scholars have argued that it is the overall constellation rather than the presence of particular individual factors that determine success [43]. The more informal networking and knowledge transfer becomes, the more organic and self-sustaining it is. Therefore, knowledge transfer and learning cannot be fully centrally planned. It needs to be evolving and shared between central program management and participating organizations.

Understanding how various stakeholder groups acquire and use knowledge and the relative efforts and benefits of using and producing knowledge can help facilitate knowledge transfer [42]. Our work supports the notion that this is a complex and dynamic process characterized by collaboration as well as competition involving various forms of learning [44]. The literature highlights the need for national guidance to stimulate the establishment of a learning ecosystem [42], but there is limited evidence on how this may be achieved and how health systems can organize knowledge transfer more effectively. Here, we provide a starting point for addressing these issues. For example, geographical proximity, communal needs, and technological and cultural similarity can promote interorganizational knowledge transfer, particularly where they enable existing links between organizations and individuals [45,46].

Insights from studies of learning economies in commercial settings are likely to have limited applicability to health, as contexts and underlying processes differ. For example, health system stakeholders are often motivated to exchange knowledge to contribute to public good and patient care, whereas profit is likely to motivate commercial organizations primarily. Nevertheless, there are some areas of convergence that indicate that certain underlying processes are generic. Existing research shows that knowledge transfer across organizations can be achieved using different mechanisms, including databases and codified documents (supporting the importance of the blueprinting process) [47]; workshops and meetings [48]; task forces, visits, and personnel transfers (supporting the importance of informal visits) [44,49,50]; formation of user communities [22]; and formation of alliances (supporting the importance of GDE and fast follower partnerships) [51].

Our results also support existing work highlighting the importance of informal networks and that implementation and optimization experience is difficult to codify and transfer [52-54]. Although different mechanisms vary in effectiveness [55], the most effective way to transfer tacit knowledge is through people interacting and perhaps moving between settings or establishing communities of practice [15,56,57]. Blueprints, as repositories of formal knowledge, can help lower entry costs for neophytes, but they need to be supported by complex informal networking-based approaches that promote different types of learning [58-61].

Moreover, we identified the important emerging role of various intermediaries in knowledge transfer. This intermediary role is often carried out by people, enabled by their location and attitude, rather than through formally managed planned arrangements [19,62,63]. Vendors frequently bring their users together to obtain insights into the context of use of their offerings, which helps them refine and market these [64]. Users can exploit these fora to network and share experience together, thereby securing influence over product enhancement as well as over the strategies adopted by vendors [65]. Professional groupings and some independent organizations geared toward mediating knowledge exchange are also effective forms of intermediaries, as they do not have conflicting interests and therefore do not seek to control members’ activities (allowing knowledge to flow freely) [66].

### Implications for Policy, Practice, and Research Emerging From This Work

This groundbreaking attempt to create a national digital health learning ecosystem illustrates that formal top-down interventions (such as partnering arrangements, the production of artifacts such as blueprints, funding, and coordination activities) can stimulate the beginnings of a learning ecosystem, but informal relationships (arising from these initiatives or emerging independently) are important for effectiveness and sustainability [67,68]. However, informal networks are difficult to plan, and knowledge transfer and networking cannot be anticipated. Therefore, support should seek to assist informal knowledge markets where formal means have failed. These may include promoting secondments and consultancy to promote knowledge transfer through *social learning*.

Central strategies cannot, however, guarantee that effective informal knowledge transfer will occur; therefore, there is a degree of uncertainty in relation to both intended and unintended outcomes. Policy intervention needs to be evolutionary, establishing ways to help link stakeholders with similar concerns and needs, offering tools to facilitate communication, and encouraging the activity of independent intermediaries [19,56].

### Conclusions

Interorganizational knowledge transfer was promoted by formal structures initiated through the GDE program. Informal processes play a key role in knowledge transfer, but they are highly contingent and cannot be readily promoted and sustained by conventional top-down planning structures. National mechanisms to stimulate knowledge sharing, therefore, need to be flexible to align with emerging, changing needs, and need to be sustained through informal networking driven by the mutual benefits of knowledge exchange. Benefits are most immediate and networking most readily sustained where there is strong convergence between group members in their organizational and technological setting and goals, such that the costs of learning are minimized and the benefits of learning are maximized. Recent concerted efforts to deploy digital solutions during the COVID-19 pandemic have reinforced this point [69].

The program laid the foundation for a digital health learning ecosystem. However, interpersonal knowledge transfer (eg, through networking and visits) is labor- and resource-intensive and may be difficult to scale and sustain. Knowledge transfer through circulating documents such as blueprints, although potentially scalable and low cost, is unlikely to be effective by itself. This situation calls for evolving strategic and policy frameworks, shaped by a mixture of top-down and bottom-up inputs, with a trusting relationship between those who facilitate knowledge exchanges and those involved in actively sharing and using that knowledge.

## Appendix

Multimedia Appendix 1 Number of interviews, observations, and documents collected in each case study site.

## Abbreviations

COREQ: Consolidated Criteria for Reporting Qualitative Studies
GDE: Global Digital Exemplar
HIT: Health Information Technology
NHS: National Health Service
