# Supplementary material for: Interorganizational Knowledge Sharing to Establish Digital Health Learning Ecosystems: Qualitative Evaluation of a National Digital Health Transformation Program in England
Source: J Med Internet Res. 2021 Aug 19;23(8):e23372. doi: 10.2196/23372 (PMC8414305; doi:10.2196/23372)
Supplement: Multimedia Appendix 1 [file jmir_v23i8e23372_app1.docx]

Appendix 1 – Table with numbers of interviews, observations and documents collected in each case study site

|  | **Site ID** | **Interviews** | | **Observations** | | **Documents** | | **Total** | **Interviewee Category** |
| --- | --- | --- | --- | --- | --- | --- | --- | --- | --- |
|  |  | **Broader study** | **In-depth study** | **Broader study** | **In-depth study** | **Broader study** | **In-depth study** |  |  |
| **In-depth case study sites (n=12; Sites A to M)** | **A** | 20 | 16 | 5 | 1 | 4 |  | 291 | Senior manager: 36  Clinical digital leader: 61  Non-clinical digital leader: 37  GDE programme staff: 45  Operational staff: 112 |
|  | **B** | 7 | 9 | 3 | 2 | 1 | 7 |  |  |
|  | **C** | 19 | 10 | 1 | 2 | 3 | 1 |  |  |
|  | **D** | 8 | 19 | 2 | 3 | 4 | 4 |  |  |
|  | **E** | 12 | 20 | 1 | 5 | 2 | 5 |  |  |
|  | **F** | 6 | 23 | 3 | 2 | 2 | 2 |  |  |
|  | **G** | 9 | 19 | 1 | 17 | 4 | 35 |  |  |
|  | **H** | 6 | 8 | 3 | 1 | 4 | 2 |  |  |
|  | **I** | 7 | 17 | 3 | 7 | 4 | 9 |  |  |
|  | **J** | 2 | 10 | 3 |  | 1 | 3 |  |  |
|  | **L** | 8 | 15 | 2 |  | 1 | 4 |  |  |
|  | **M** | 6 | 15 |  |  | 1 | 1 |  |  |
| **Broader case study sites (n=24; Sites 1-24)** | **1** | 4 |  |  |  | 3 |  | 166 | Senior manager: 20  Clinical digital leader: 44  Non-clinical digital leader: 48  GDE programme staff: 31  Operational staff: 23 |
|  | **2** | 10 |  | 2 |  | 3 |  |  |  |
|  | **3** | 6 |  | 1 |  | 4 |  |  |  |
|  | **4** | 10 |  |  |  | 8 |  |  |  |
|  | **5** | 7 |  | 3 |  | 3 |  |  |  |
|  | **6** | 7 |  | 1 |  | 4 |  |  |  |
|  | **7** | 7 |  | 1 |  | 6 |  |  |  |
|  | **8** | 6 |  |  |  | 8 |  |  |  |
|  | **9** | 9 |  | 1 |  | 8 |  |  |  |
|  | **10** | 10 |  | 1 |  | 2 |  |  |  |
|  | **11** | 7 |  |  |  | 10 |  |  |  |
|  | **12** | 10 |  |  |  | 8 |  |  |  |
|  | **13** | 7 |  |  |  | 4 |  |  |  |
|  | **14** | 7 |  |  |  | 10 |  |  |  |
|  | **15** | 7 |  |  |  | 9 |  |  |  |
|  | **16** | 4 |  | 1 |  | 4 |  |  |  |
|  | **17** | 7 |  | 2 |  | 5 |  |  |  |
|  | **18** | 5 |  | 1 |  | 5 |  |  |  |
|  | **19** | 4 |  |  |  | 9 |  |  |  |
|  | **20** | 5 |  |  |  | 9 |  |  |  |
|  | **21** | 11 |  | 1 |  | 8 |  |  |  |
|  | **22** | 5 |  | 2 |  | 7 |  |  |  |
|  | **23** | 8 |  | 2 |  | 3 |  |  |  |
|  | **24** | 3 |  |  |  | 1 |  |  |  |
|  | **Total** | 276 | 181 | 46 | 40 | 172 | 73 |  |  |
|  |  | 457 | | 86 | | 245 | |  |  |
